# Pleural fluid secondary to pulmonary cryptococcal infection: a case report and review of the literature

**DOI:** 10.1186/s12879-019-4343-2

**Published:** 2019-08-12

**Authors:** Yuan Zhang, Sean X. Zhang, Julie Trivedi, Adam D. Toll, Julie Brahmer, Russell Hales, Sarah Bonerigo, Mingying Zeng, Huiping Li, Rex C. Yung

**Affiliations:** 1 Department of Pulmonary and Critical Care Medicine, Johns Hopkins University School of Medicine, 1830 East Monument Street, 5th Floor, Baltimore, MD 21205 USA; 2 Department of Pathology, Johns Hopkins University School of Medicine, Baltimore, USA; 3 Department of Medicine, Division of Infectious Diseases, Johns Hopkins University School of Medicine, Baltimore, USA; 4 Department of Medical Oncology, Johns Hopkins University School of Medicine, Baltimore, USA; 5 Department of Radiation Oncology and Molecular Radiation Sciences, Johns Hopkins University School of Medicine, Baltimore, USA; 6 Department of Respiratory Medicine, Shanghai Pulmonary Hospital, Tongji University School of Medicine, 507 Zheng Min Road, Shanghai, 200433 China

**Keywords:** Pulmonary cryptococcosis, Pleural effusion, Crytococcal antigen, Lateral flow assay

## Abstract

**Background:**

Pulmonary Cryptococcosis (PC) is diagnosed with increasing incidence in recent years, but it does not commonly involve the pleural space. Here, we report a HIV-negative case with advanced stage IIIB non-small cell lung cancer (NSCLC) treated with radiation therapy presented with dyspnea, a new PET-positive lung mass and bilateral pleural effusion suspecting progressive cancer. However, the patient has been diagnosed as pulmonary cryptococcal infection and successfully treated with oral fluconazole therapy.

**Case presentation:**

A 77-year-old male with advanced stage non-small cell lung cancer treated with combined chemo-radiation therapy who presented with progressive dyspnea, a new PET-positive left lower lobe lung mass and bilateral pleural effusions. Initial diagnostic thoracentesis and bronchoscopy yielded no cancer, but instead found yeast forms consistent with cryptococcal organisms in the transbronchial biopsies of the left lower lobe lung mass. Subsequent to this, the previously collected pleural fluid culture showed growth of *Cryptococcus neoformans*. The same sample of pleural effusion was tested and was found to be positive for crytococcal antigen (CrAg) by a lateral flow assay (LFA). The patient has been treated with oral fluconazole therapy resulting in gradual resolution of the nodular infiltrates.

**Conclusion:**

PC should be considered in immunosuppressed cancer patients. Additionally, concomitant pleural involvement in pulmonary cryptococcal infections may occur. The incidence of false positive ^18^FDG-PET scans in granulomatous infections and the use of CrAg testing in pleural fluid to aid in diagnosis are reviewed.

## Background

Pulmonary cryptococcosis (PC) is diagnosed with increasing incidence in recent years in both immunocompromised and immunocompetent patients, but it does not commonly involve the pleural space. The worldwide incidence of pulmonary cryptococcal infection has been increasing over the past decades in both HIV infected and in non-HIV patients [1–4]. Although PC occurs in immunocompetent hosts, the increase in hematologic stem-cell and solid organ transplant patients, the expanded use of immunosuppressive drugs in inflammatory disorders, and cytotoxic chemo-radiation therapies in cancer patients have increased its prevalence.

Here, we report a HIV-negative case with advanced stage IIIB non-small cell lung cancer (NSCLC) treated with radiation therapy presented with dyspnea, a new PET-positive lung mass and bilateral pleural effusion suggestive of progressive cancer. However, the patient has been diagnosed as PC and was successfully treated with oral fluconazole therapy.

## Case presentation

**Fig. 1:**
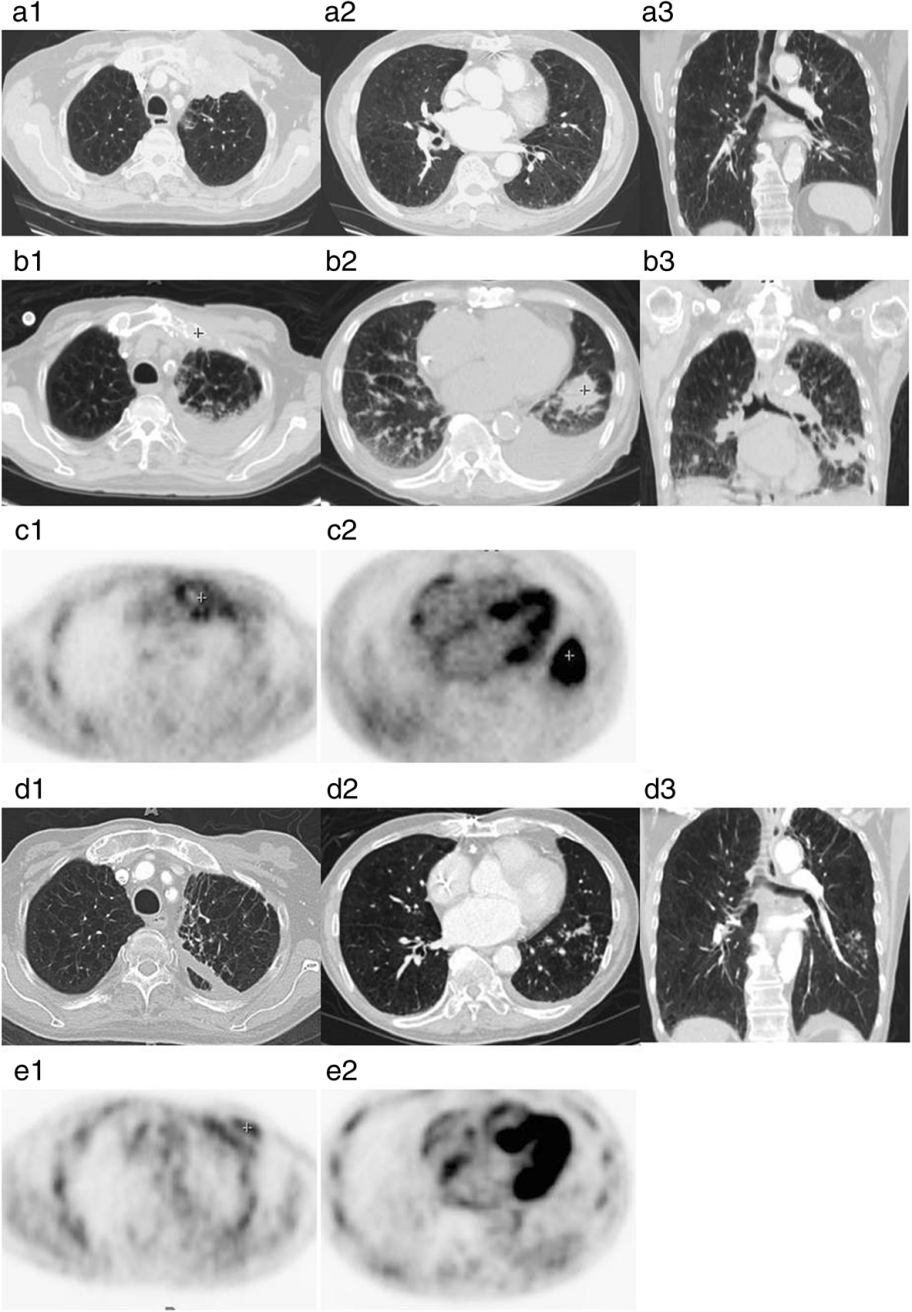
**a1**-**a3**: Baseline CT images of the left upper chest wall tumor, a large 6 cm × 9 cm LUL mass invading through the left anterior chest wall into ribs as (four months prior to pleural effusion); **b1**-**b3**: Response in the left upper lobe chest wall mass, but appearance of a new LLL nodular mass as well as pleural effusion (March); **c1**-**c2**:^18^FDG-PET image demonstrated intense FDG activity fusing to 3.3 × 4 cm left lower lobe mass with SUV max 9.9, but minimal left upper lobe chest wall uptake (March); **d1**-**d3**:Chest CT scan post-fluconazole therapy (five months post-fluconazole therapy) showed that Left lower lobe mass has significantly diminished in size. Right pleural effusion had resolved; **e1**-**e2**: 18FDG-PET image follow-up post PC therapy shows resolution of LLL uptake, with some minimal chest-wall uptake (eight months post-fluconazole therapy)

A 77-year-old Chinese American male with stage IIIB (T4N2M0) NSCLC was referred for evaluation and management of progressive dyspnea in March. His past medical history included a 120 pack years smoking history and severe underlying chronic obstructive pulmonary disease (COPD). Four months prior to this presentation, he developed non-anginal anterior chest pain. A large 6 cm × 9 cm lung mass invading through the left anterior chest wall into ribs and manubrium was biopsied and found to be a squamous cell cancer (Fig. 1a). The patient underwent definitive radiation therapy with a good local response, but he had poor systemic tolerance to one course of the chemotherapy given post radiation. Even though he had severe COPD and pre-existing severe diffuse atherosclerotic vascular disease (coronary artery disease, status post coronary artery bypass, bilateral carotid endarterectomies, hypertension, hypercholesterolemia and hyperlipidemia), he neither had any significant exertional dyspnea nor history of congestive heart failure prior to the cancer diagnosis and initiation of cancer therapy. A follow-up CT scan was performed in March (Fig. 1b). At the time of his progressive dyspnea, shrinkage of the left chest wall mass and left upper lobe infiltrates was demonstrated, but appearence of a new 3.9 cm × 4 cm left lower lobe (LLL) mass outside the previous radiation portal and new bilateral pleural effusions were observed. ^18^FDG-PET scan (Fig. 1c) showed intense uptake (SUVmax 9.9) fusing to the new LLL mass, while the original left upper chest wall tumor site only revealed moderate uptake of SUV 2.6 consistent with post treatment changes.

On review of systems, the patient denied angina, orthopnea nor paroxysmal nocturnal dyspnea that helped to rule out congestive heart failure. He had a non-productive cough and didn’t have hemoptysis. He also denied fever, chills or night sweats. Physical examination revealed dullness to percussion at the lung bases, consistent with bilateral pleural fluid. He had trace lower extremity edema. Other pertinent studies included a cardiac echocardiogram from two months prior, when the patient had a left ventricular ejection fraction (LVEF) of 45–50%, and trace tricuspid regurgitation with an estimated right ventricular systolic pressure (RVSP) of 50 mmHg.

Left sided thoracentesis was performed twice for diagnostic purposes. The first tap removed 1000 ml of orange-red sero-sanguinous fluid, that on chemical analysis suggested a transudate as the pleural protein was only 3.0 with a serum protein of 6.2 g / dl (ratio < 0.5) and the pleural LDH of 122 is also < 0.6 of the serum LDH. The pH was 7.40 and pleural glucose was 104. Bacterial culture was negative and cytology revealed reactive mesothelial cell, histiocytes and lymphocytes. The pleural fluid cell count revealed a lymphocytosis of 596 /cu mm, neutrophils of 308 / cu mm while the peripheral WBC of 8370 / cu mm showed a lymphopenia with absolute lymphocyte count of 320 / cu mm. The fluid rapidly re-accumulated and on March 30th, only 18 days later, another 1400 cm^3^ of hemorrhagic effusions was removed but this time the chemistry was consistent with exudate with a pleural protein of 3.6 g/dl. Initial cultures for bacteria, fungi and mycobacterium were negative. Repeat cytology revealed reactive mesothelial cells, histiocytes, lymphocytes and blood without malignant cells.

**Fig. 2:**
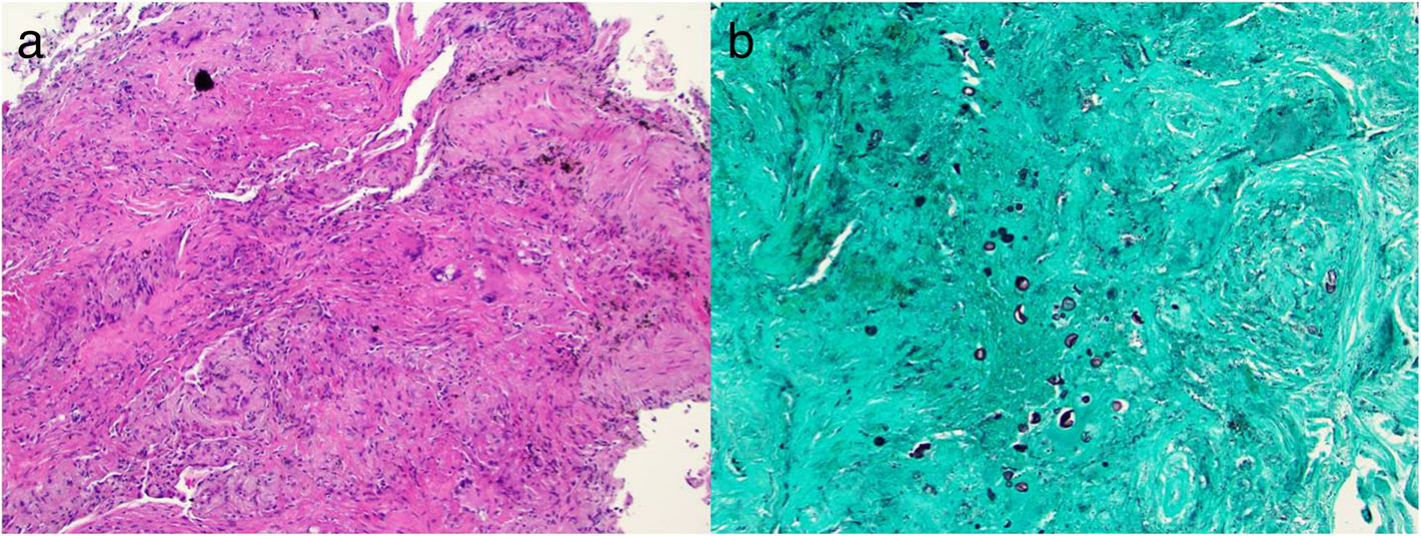
Pathology H & E (**a**) and silver staining (**b**) demonstrating granulomatous inflammation and fungal organisms consistent with *Cryptococcus neoforman*

Due to the continued lack of a treatable diagnosis, diagnostic bronchoscopy was performed on April 6th. Endobronchial ultrasound (EBUS) guided transbronchial needle sampling of regional lymph nodes and the LLL mass were negative for the tumor. However, transbronchial forceps biopsy of the LLL mass showed granulomatous pneumonitis with fungal elements consistent with capsule deficient *Cryptococcus* on H&E and gomori methenamine silver stain (Fig. 2). Bronchoalveolar lavage (BAL) sent for pan-culture did not grow any pathogens, and the BAL galactomannan was negative at 0.36 (cutoff < 0.50). Subsequently, the pleural fluid collected on March 30th grew *Cryptococcus neoformans* after 10 days of culture.

The patient’s pulmonary cryptococcal infection with pleural involvement was probably attributed to his cancer and the treatment. His HIV test was negative. A serum Cryptococcal antigen (CrAg) was negative. He did not have neurologic symptoms, and a baseline MRI of the brain done at the time of initial cancer diagnosis was negative except for findings suggestive of chronic small vessel ischemic disease. Because pleural involvement was considered extrapulmonary spread of the infection, and his present CD4 lymphopenia was an ongoing risk for dissemination, he underwent a lumbar puncture with negative cerebrospinal fluid (CSF) findings (including negative CSF CrAg). Serum galactomannan level was negative at 0.08. Upon retrospective review, the patient had a baseline lymphopenia of 950 / cu mm prior to the initial radiation and chemotherapy. The lymphopenia had never recovered with absolute lymphocyte counts (ALC) between 400 and 600 / cu mm. A T-cell subset checked at the time of the first thoracentesis revealed a CD 4+ cell percent of 24.3% (normal range 32–68%) and absolute CD4+ count of 112/cu mm (normal range 458–1344 /cu mm). Pleural fluid collected from the first thoracentesis on March 12th was analyzed for the presence of CrAg. It was negative by enzyme-linked immunosorbentassays (ELISA), but was positive by lateral flow assay (LFA).

Fluconazole therapy, dose adjusted for renal dysfunction at 400 mg PO q24 was started on April 9th. Subsequent follow-up for 10 months after initiation of the treatment showed progressive clinical improvements with resolution of dyspnea, cough, recovery of appetite and radiographic improvement in chest imaging (Fig. 1d and e). He finally came to us in April of 2015 for a checkup of his supraclavicular lymphadenopathy and chest CT scan showing new nodules of bilateral lungs in April of 2015. We performed fine needle aspiration and the reports showed malignancy. Hence, the patient refused to undergo further management and he passed away that year after six months.

## Discussion and conclusions

This case is noteworthy for the following: (1) Uncommon but possibly under-recognized pleural involvement in PC infections; (2) testing of pleural fluid for CrAg; (3) Nonspecific ^18^ FDG uptake in PET imaging caused by unsuspected opportunistic infections or other inflammatory conditions that can lead to false positive misdiagnosis of lung cancer dissemination.

The radiologic presentation of PC varies with patterns dependent upon host immune function. Immunocompetent patients generally present radiographically with single nodule, or sometimes multiple nodules. On the other hand, in immunocompromised cases, pneumonic or mixed nodular-pneumonic patterns, or cavitatary lesions combined with pulmonary infiltrates are more common [5–7]. Pleural effusions although reported, are uncommon in PC cases [7–12]. Pleural effusions due to *Cryptococcus neoformans* infection were first reported in the English literature in 1941 [8], with sporadic cases reported [9–11] until 1980 when Young and co-workers presented only 2 of their own cases but they were reviewed together with 28 cases previously reported in the literature [12]. In our recent report of 76 Chinese patients with PC [7], only 5 cases (6.58%, 5/76) had associated pleural effusion, almost equally divided between immunocompetent and immunocompromised patients (2 vs. 3, respectively).

In addition, our patient developed a new PET-positive focal lung mass with intense ^18^FDG uptake (SUV max 9.9) that is also consistent with metastatic disease. Because ^18^FDG is a non-specific tumor-imaging agent, ^18^FDG -PET scans performed for the evaluation of focal lung lesions suspicious for malignancies will yield false positive results in a number of granulomatous infectious and inflammatory conditions due to non-specific glucose uptake. The specificity of a “positive” scan for malignancy is lowered from mid-80% to 40–60% range in regions of endemic granulomatous fungal and mycobacterial infections [13–20]. Specifically in PC, in our review of 76 patients with pathologically proven PC [7], the initial clinical misdiagnosis rate for lung cancer was 30.26% (23/76), and this was largely attributable to a high ^18^FDG uptake. 46 of the 76 cases with focal PC had a ^18^F-FDG-PET scan, and 60.87% (28/46) showed high degree of abnormal uptake in the lung lesions (SUV > 2.5), which typically indicates malignancy. Therefore, we would like to highlight that although 18F-FDG-PET has a good sensitivity in identifying metabolically active thoracic malignancies, it may be falsely positive secondary to granulomatous disease. Findings of unclear significance on PET after cytotoxic therapy underscore the importance of confirmatory tissue biopsy. In this case the combination of histologic findings, special stains, and the ultimate proof of a positive fungal culture from the pleural fluid provided a diagnosis and allowed initiation of optimal therapy.

A positive fungal culture also plays a key role in the management of PC cases. According to the review of PC cases with pleural effusions, cultures of pleural effusion for *Cryptococcus* were positive in 42.31% (11/26) of cases in which this were recorded [12]. Our present case also showed positive *Cryptococcal neoformans* culture, and interestingly only of pleural effusion but not of the BAL fluid. It is now thought that *Cryptococcal neoformans* is the most common risk for cryptococcosis in immunocompromised patients especially acquired immune deficiency syndrome (AIDS), whereas infections caused by *C. gattii* are more often reported in immunocompetent patients with undefined risk than in the immunocompromised.

Based on clinical guidelines, oral fluconazole therapy (400 mg, 6–12 months) was recommended for non-meningeal cryptococcosis in immunosupressed patients with mild-moderate symptoms patients. Previous studies also reported that the outcome of PC is often better in localized diseases than that of disseminated diseases [12].

In addition to fungal culture, the cryptococcus antigen (CrAg) test is considered an effective non-invasive diagnostic tool of PC [21]. The role of CrAg test in serum and CSF is well accepted [21, 22]. In literature review, the sensitivity / specificities for CrAg is as high as 98%/98% in serum, and 100%/99% in CSF [21]. As for testing CrAg in pleural effusion, apart from our case, there were 9 published cases in which CrAg test was recorded in the English literature since 1978 [11, 12, 23–29] As listed in Table 1 [30–32], 8 of the 10 cases (80) % showed positive CrAg in the pleural fluid. 6 out of 10 showed positive pleural PC culture. Two cases [11, 29] that were negative for pleural fluid CrAg were also negative for culture. Two cases [25, 27] were negative by culture but positive for CrAg. Positive pleural fluid CrAg per-se does not predict a worse prognosis (5/8 PE CrAg positive cases that were also culture positive were localized), however when there is a discordance of positive results, specifically when the pleural CrAg was positive but the pleural fluid culture was negative, the two cases were disseminated. The significance of this observation is unclear in a small collection of case reports, and more studies are needed to determine whether finding an antigen in dispersed sources in the absence of culture positivity could portend dissemination. The newly released CrAg test method of lateral flow assay (LFA) has been found to be more sensitive (unpublished data) and requires minimal laboratory infrastructure [30]. In our case, pleural fluid was tested for CrAg by two methods, the ELISA test was negative, but the LFA test was positive. Recent study targeting HIV+ patients with CrAg screening and pre-emptive fluconazole therapy in have demonstrated cost-saving to the health care systems, hence its integration into routine HIV care for persons with CD4 < 100 cells per microliter is recommended [30]. Our patient does not have HIV, but did have baseline deficits in his cell-mediated immunity with lymphopenia and a low CD4 count. The findings in our case and in the literature suggests that an opportunistic infection due to atypical non-pyogenic infections, including PC should be considered in patients with serious underlying diseases compromising the subject’s immune function. Future studies are needed to evaluate CrAg screening in non-HIV but at-risk patients and a sensitive CrAg test may be included in the evaluation of idiopathic pleural effusions in such cases. Our patient had lung histology consistent with PC, and yet BAL culture was negative for fungi. Whether there is a role of CrAg testing for BAL and by which technique it can be done is another topic for further investigation.

**Table 1:** Summary of pleural effusion (PE) tested with CrAg in PC cases in the literature

Year	Author	Age/gender	Localized or Disseminated	Predisposing Conditions	Lympopenia	Culture for *Cryptococcus neoformans*					CrAg			Pulmonary Lesions	Treatment	Out-come
						PE	BAL	Serum	CSF	Sputum	PE	Serum	CSF			
1978	Gross	66/M	Disseminated	Steroids	Yes	**–**	+	NR	NR	NR	**–**	+	–	Left side PE and LLL infiltrate	Amphotercin B; 5-fluorocytosine; transfer factor; surgery	CR
1980	Young case 1	42/M	Localized	Diabetes mellitus, Chronic renal failure	NR	**+**	NR	–	–	NR	**+**	–	–	Right side PE	Amphotercin B; 5-fluorocytosine	CR
	case2	66/F	Localized	Accurate renal failure	NR	**+**	NR	–	+	NR	**+**	+	+	Bilateral PE	Amphotercin B	CR
1990	Conces	53/F	Disseminated	Renal transplant	NR	**+**	NR	NR	–	NR	**+**	NR	–	Right side PE	Amphotercin B;	NR
1998	Fukuchi	52/F	Localized	Chronic renal failure; Rheumatoid arthritis; Steroids	No	**+**	NR	NR	–	NR	**+**	+	–	Left side PE and LLL infiltrate	Amphotercin B; 5-fluorocytosine; Fluconazole	CR/PR
1999	Wong	30/F	Disseminated	None	NR	**–**	NR	–	+	NR	**+**	NR	+	Bilateral PE; LLL cavitating infiltrate	Amphotercin B; 5-fluorocytosine; Fluconazole	CR
2007	Jain et al.	50/M	Localized	HIV-positive	Yes	**+**	NR	NR	NR	NR	**+**	NR	NR	Left side PE	Fluconazole	CR
2008	Kamiya et al.	83/M	Disseminated	Myelodysplastic syndrome	Yes	**–**	NR	+	–	NR	**+**	+	+	Bilateral PE and RLL infiltrate	Fosfluconazole; Lyposomal-amphotercin B	PR/Died^a^
2009	Kamminga et al.	47/F	Disseminated	Thymoma undering chemotherapy	NR	**–**	–	–	+	NR	**–**	–	+	Right side PE; Thymoma in the left hemithorax	Amphotercin B; 5-fluorocytosine; Fluconazole	CR
2012	Present case	77/M	Localized	NSCLC Stage IIIB with Radio-chemotherapy; COPD	Yes	**+**	–	ND	–	–	**+**	–	–	Left side PE and LLL infiltrate	Fluconazole, oral 400 mg/d	CR
2015		63/M	Localized	renal transplant recipient on an immunosuppressive regimen	No	NR	ND	+	ND	NR	ND	ND	ND	Left-sided pleural effusion with compressive atelectasis of LLL	amphotericin B liposome combined with 5-flucytosine and voriconazole for first 11 days, then amphotericin B liposome combined with 5-flucytosine sustained to 8 weeks, after that changed to fluconazole for maintenance	PR
2018	Kushima et al.	80/M	Localized	Steroids treatment due to rheumatoid arthritis, then developed pulmonary tuberculosis and cryptococcal pleuritis	No	**+**	NR	NR	NR	NR	ND	+	ND	Bilateral PE and Consolidation in LUL and a nodule in the LLL	Anti-tuberculosis and anti-fungal agents	PR/Died^a^

*NR* Not recorded, *CR* Cured/complete Response, *PR* Partial Response

^a^*Cryptococcus neoformans* disappeared from the right pleural effusion, and his condition improved temporarily by continuing fosfluconazole after discontinuing L-AMB because of renal dysfunction. However, the patient gradually became malnourished, and died of respiratory failure after a complication of aspiration and renal failure regardless of antibiotic therapy

Pleural involvement by pulmonary cryptococcal infections is under-appreciated, and can occur in non-HIV patients who are not manifesting typical signs of an infection. In patients with underlying malignancies with findings on imaging suggestive of recurrent or progressive disease, abnormal ^18^FDG-PET uptake findings require confirmatory tissue biopsy and culture. When biopsy results are non-specific, additional studies for invasive granulomatous infections, including pulmonary cryptococcosis should be considered. Where indicated, evaluation of pleural effusion should include sending specimen for a sensitive CrAg assay and for fungal culture. Prompt diagnosis and guidelines-directed therapy will improve outcomes in patients who may otherwise have stable underlying cancer, but may succumb to infectious complications.

## Data Availability

Not applicable (no datasets were generated or analyzed during the current study).

## Abbreviations

AIDS: Acquired immune deficiency syndrome
BAL: Bronchoalveolar lavage
COPD: Chronic obstructive pulmonary disease
CrAg: Crytococcal antigen
CSF: Cerebrospinal fluid
EBUS: Endobronchial ultrasound
ELISA: Enzyme-linked immunosorbentassays
LFA: Lateral flow assay
LLL: Left lower lobe
LVEF: Left ventricular ejection fraction
PC: Pulmonary Cryptococcosis NSCLC: Non-small cell lung cancer
RVSP: Right ventricular systolic pressure
